# Low-Temperature Ammonia Synthesis on Iron Catalyst with an Electron Donor

**DOI:** 10.1021/jacs.2c13015

**Published:** 2023-03-30

**Authors:** Masashi Hattori, Natsuo Okuyama, Hiyori Kurosawa, Michikazu Hara

**Affiliations:** Laboratory for Materials and Structures, Tokyo Institute of Technology, 4259 Nagatsuta, Midori-ku, Yokohama 226−8503, Japan

## Abstract

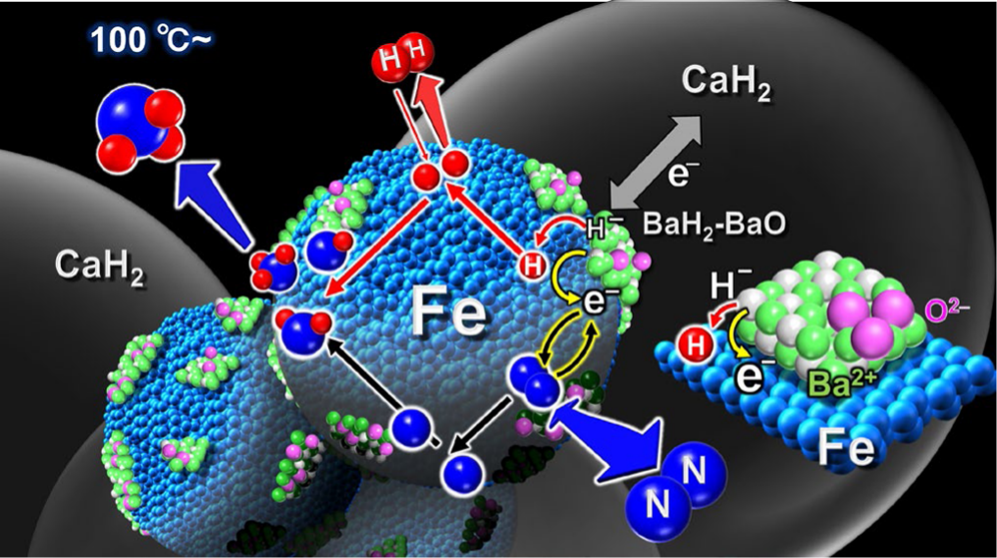

Haber–Bosch process produces ammonia to provide food for over 5 billion people; however, it is currently required to be produced without the use of fossil fuels to reduce global CO_2_ emissions by 3% or more. It is indispensable to devise heterogeneous catalysts for the synthesis of ammonia below 100–150 °C to minimize the energy consumption of the process. In this paper, we report metallic iron particles with an electron-donating material as a catalyst for ammonia synthesis. Metallic iron particles combined with a mixture of BaO and BaH_2_ species in an appropriate manner could catalyze ammonia synthesis even at 100 °C. The iron catalyst revealed that iron can exhibit a high turnover frequency (∼12 s^–1^), which is over an order of magnitude higher than those of other transition metals used in highly active catalysts for ammonia synthesis. This can be attributed to the intrinsic nature of iron to desorb adsorbed hydrogen atoms as hydrogen molecules at low temperatures.

## 1 Introduction

**Figure 1:**
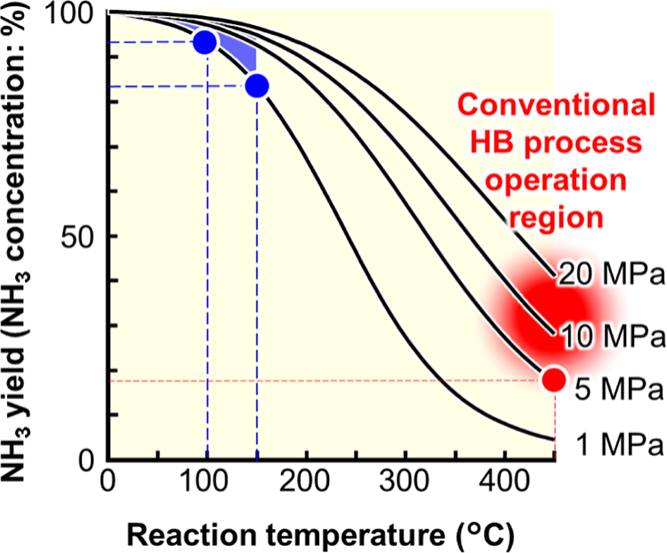
Correlation of ammonia yield with temperature and pressure.

The low ammonia yield has been a significant issue in the Haber–Bosch (HB) process for over 100 years. The maximum ammonia yield at equilibrium decreases with increasing reaction temperature in the HB process (Figure 1). The operating temperature for the iron-based catalyst used in the present process exceeds 400 °C, which requires pressurization of the process to ∼20 MPa to obtain a yield of 30–40% at most.^1−6^ However, any catalyst that could allow the operating temperature to be decreased below 100–150 °C would significantly increase the ammonia yield. Therefore, this issue has pushed recent research toward low-temperature ammonia synthesis.

Most of the recently reported highly active catalysts use Ru, Co, and Ni as reaction sites because these can be easily deposited on supports as highly dispersed metal nanoparticles that exhibit high catalytic performance.^7−11^ While we have also discovered a unique Ru-based heterogeneous catalyst to synthesize ammonia from H_2_ and N_2_, even below 100 °C,^12^ we have questioned whether catalysts that use these transition metals have significant potential to increase the catalytic activity at the desired reaction temperature of ≤100–150 °C from the perspective of the H_2_ adsorption/desorption equilibrium. The dissociative adsorption of H_2_ and H_2_ desorption from the resulting adsorbed H atoms (H adatoms) are in equilibrium on all transition metal surfaces in the synthesis of ammonia as well as N_2_ adsorption/desorption. It is well known for Ru catalysts that adsorption of H adatoms onto the transition metal surfaces is preferential so that the H_2_ adsorption/desorption equilibrium shifts toward H_2_ adsorption, which decreases ammonia formation by the decrease in adsorption sites for N adatoms.^13,14^ Such hydrogen-poisoning can affect all transition metals used in the synthesis of ammonia. Attributing hydrogen-poisoning to the bond strength between H adatoms and the surface transition metal atoms, it is anticipated that this effect is enhanced with a decrease in ammonia synthesis temperature because a larger amount of H adatoms would be more tightly adsorbed on the transition metal surfaces with a decrease in temperature. As a result, hydrogen-poisoning would be a major obstacle to the desirable low-temperature ammonia synthesis over catalysts that employ transition metals as the active sites.

In the present study, we have again focused on iron used as the reaction site since the beginning of the HB process. Iron has been regarded as a classical transition metal that is inferior to other transition metals for ammonia synthesis. Several catalysts that use other transition metals exhibit much higher catalytic performance for ammonia synthesis than iron-based catalysts used in the present HB process.^8,12^ In addition, no effective ways to use iron for ammonia synthesis at low temperatures as low as 100 °C have been found to date. On the other hand, ammonia synthesis over iron-based catalysts has not been reported to be strongly influenced by hydrogen-poisoning. This suggests that the H_2_ adsorption/desorption equilibrium on iron surfaces is shifted more toward H_2_ desorption, and thus a decrease in the H adatom concentration than with other transition metals. Therefore, iron may be used for ammonia synthesis while preventing hydrogen-poisoning, even at low temperatures, if iron can be combined with an appropriate promoter in an appropriate manner. The use of ubiquitous, abundant, and inexpensive iron is also a significant advantage with respect to the environment and economy.

## 2 Methods

### 2.1 Preparation of Iron Particles Loaded with and without BaH_2_-BaO (BaH_2_-BaO/Fe/CaH_2_, Fe/CaH_2_)

BaH_2_-BaO/Fe/CaH_2_ and Fe/CaH_2_ were typically prepared by heating a mixture of Ba(NO_3_)_2_-impregnated α-Fe_2_O_3_ and CaH_2_ particles and a mixture of Fe_2_O_3_ and CaH_2_ particles, respectively, in a flow of H_2_. 7.0 g of Fe(NO_3_)_3_·9H_2_O (Kanto Chemical) was dissolved in 30 mL of distilled water, which was then removed from the solution by rotary evaporation at 70 °C. The remaining red solid was heated at 300 °C for 10 h in air to yield α-Fe_2_O_3_ powder. For the preparation of BaH_2_-BaO/Fe/CaH_2_, 0.20 g of α-Fe_2_O_3_ and 0.052 g of Ba(NO_3_)_2_ (High Purity Chemicals) were stirred in 10 mL of distilled water for 30 min, and the water was then evaporated at 70 °C. The resultant Ba(NO_3_)_2_-impregnated α-Fe_2_O_3_ was heated at 300 °C for 10 h in air. In an Ar-filled glovebox, 0.023 g of Ba(NO_3_)_2_-impregnated α-Fe_2_O_3_ and 0.077 g of CaH_2_ were mixed in an alumina mortar and transferred into a stainless steel fixed-bed reactor for ammonia synthesis (see Supporting Information 1.1 and Figure S1). The reactor was removed from the Ar-filled glovebox to the atmosphere and connected to a flow reaction system without exposure of the mixture in the reactor to the atmosphere. The mixture was heated in a flow of H_2_ (45 mL min^–1^) at 300 °C for 2 h, which resulted in BaH_2_-BaO/Fe/CaH_2_. Inductively coupled plasma (ICP) spectroscopy analysis indicated that the atomic ratio of Fe/Ba/Ca in the catalyst was 12.5:1.0:100.0. Fe/CaH_2_, *i.e.*, metallic iron particles without BaH_2_-BaO were obtained from a mixture of 0.019 g of α-Fe_2_O_3_ and 0.081 g of CaH_2_ by the same preparation procedure as that for BaH_2_-BaO/Fe/CaH_2_. The Fe/Ca atomic ratio in the resultant catalyst was 12.1:100.0. The size of the metallic iron particles in the catalysts was 20–40 nm (mean particle size: 26 nm).

### 2.2 Preparation of Ru or Iron Nanoparticles Deposited on BaH_2_-BaO (Ru/BaH_2_-BaO, Fe/BaH_2_-BaO)

According to a previous report,^15^ Ru/BaH_2_-BaO and Fe/BaH_2_-BaO were prepared from a mixture of 3 mol% BaO (Kojundo Chemical) and 97 mol% CaH_2_ with Ru(acac)_3_ or Fe(acac)_2_ corresponding to 10 wt % Ru or Fe. The mixture was prepared and transferred into the stainless steel fixed-bed reactor in an Ar-filled glovebox. The reactor was connected to the flow reaction system, as described in Section 2.1, and the mixture was heated at 260 °C in a flow of H_2_ (2.5 mL min^–1^). After 2 h, the samples were heated at 340 °C for 10 h in a flow of H_2_ (2.5 mL min^–1^). In the resultant materials, transition metal nanoparticles were deposited on the BaH_2_-BaO phase formed on large CaH_2_ particles (several micrometers).^15^ The transition metal particle sizes (Ru: 2–7 nm (mean particle size: 4 nm), Fe: 2–8 nm (mean particle size: 4 nm)) in the catalysts (Ru/BaH_2_-BaO, Fe/BaH_2_-BaO) were smaller than those (Fe: 20–40 nm (mean particle size: 26 nm)) of the catalysts (BaH_2_-BaO/Fe/CaH_2_, Fe particles) prepared by the method described in Section 2.1. ICP analysis indicated that the Ru/Ba/Ca and Fe/Ba/Ca atomic ratios for the Ru/BaH_2_-BaO and Fe/BaH_2_-BaO catalysts were 4.9:3.2:100.0 and 8.9:3.1:100.0, respectively.

### 2.3 Evaluation of Catalytic Performance (See Supporting Information 1.1 and Figure S1)

Ammonia synthesis over each catalyst was examined in a stainless steel fixed-bed reactor (catalyst; 0.1 g) at 300 °C under a flow of N_2_–H_2_ (N_2_ and H_2_ >99.9999%, N_2_/H_2_ = 1:3, 60 mL min^–1^, weight hourly space velocity (WHSV): 36 000 mL g cat^–1^ h^–1^) at 0.9 MPa. After no increase or decrease in activity was observed for over 20 h, the catalyst was cooled down to below 20 °C in a flow of N_2_ at a flow rate of 60 mL min^–1^ and then held under this flow for 5 h. After no ammonia formation was confirmed, the catalyst was heated at specific temperatures in a flow of N_2_–H_2_. The ammonia produced was trapped in 5 mM H_2_SO_4_ aqueous solution, and the amount of NH_4_^+^ generated in the solution was estimated using an ion chromatograph (LC-2000 plus, Jasco) equipped with a thermal conductivity detector. The rate of ammonia formation was repeatedly measured more than three times after the ammonia formation rate remained constant for over 1 h. It was verified that the measured rate of ammonia formation had an error of less than 5%.

Time of flight (TOF) was calculated from the ammonia formation rate, and the number of surface zero-valent transition metal atoms (*N*_s_) was estimated on the basis of CO chemisorption values, assuming spherical metal particles. *N*_s_ for each tested catalyst was measured by CO-pulse chemisorption (BELCAT-A, BEL, Japan) at 50 °C using a He flow of 30 mL min^–1^ and pulses of 0.09 mL (9.88% CO in He).^7^ Prior to these measurements, the catalyst after the reaction was heated with flowing He (50 mL min^–1^) at 300 °C for 1 h. The stoichiometry of the transition metal/CO was assumed to be 1. *N*_s_ for BaH_2_-BaO/Fe/CaH_2_ was estimated to be 7.2 × 10^16^ atoms g^–1^ from CO-pulse chemisorption experiments. The amount of saturated CO adsorption on the iron catalyst was also measured using a closed gas circulation and evacuation system. There was no difference in *N*_s_ between these two methods.

In the estimation of each reaction order in the rate equation (*r* = *kP*H_2_^α^*P*N_2_^β^*P*NH_3_^γ^), the reaction order of NH_3_ (γ) is estimated by measuring the NH_3_ synthesis rates with varying total gas flow at constant H_2_ and N_2_ partial pressures.^16^ The reaction orders for H_2_ (α) and N_2_ (β) were estimated by the correlations of log(*r*)−γ log(*P*NH_3_) with log(*P*H_2_) and log(*P*N_2_),^16,17^ respectively.

### 2.4 ^14^N_2_-^15^N_2_ Isotropic Exchange Reaction

N_2_ isotopic exchange was examined in a U-shaped glass reactor connected with a closed gas-circulation system. A mixture of ^15^N_2_ and ^14^N_2_ (total pressure: 20.0 kPa, ^15^N_2_/^14^N_2_ = 1:4) was adsorbed on the catalyst without circulation at the reaction temperature until adsorption/desorption was in equilibrium. The change in the composition of circulating gas was monitored by a quadrupole mass spectrometer (M-101QA-TDM, Canon Anelva Co.). The *m*/*z* = 28, 29, and 30 signals were monitored as a function of time to follow the exchange.

The formation rates for ^15^NH_3_ and ^14^NH_3_ over the tested catalysts were estimated under 0.1 MPa (H_2_/N_2_ = 3:1, WHSV: 36 000 mL g cat^–1^ h^–1^), although ammonia synthesis over the tested catalysts in this study was typically measured under 0.9 MPa. After ^14^NH_3_ synthesis from ^14^N_2_ at each reaction temperature, ^14^N_2_ was switched to ^15^N_2_ (98 atom% ^15^N, Sigma-Aldrich), and ^15^NH_3_ was synthesized at each reaction temperature. The ^14^NH_3_ or ^15^NH_3_ produced were trapped in a 5 mM H_2_SO_4_ aqueous solution, and the solution was then analyzed using ion chromatography.

### 2.5 Characterization

Powder X-ray diffraction (XRD; Miniflex600C, Rigaku) patterns were obtained using Cu Kα radiation. Nitrogen adsorption–desorption isotherms were measured at −196 °C with a surface-area analyzer (BELSORP-mini II, MicrotracBEL) to estimate the Brunauer–Emmett–Teller (BET) surface areas. The morphologies of the samples were observed using high-angle annular dark-field scanning transmission electron microscopy (HAADF-STEM) and energy dispersive X-ray spectroscopy (EDX; JEM-ARM 200F, Jeol). H_2_ and D_2_-temperature-programmed desorption (TPD) profiles were measured by heating (1 °C min^–1^) a sample (ca. 100 mg) in a flow of Ar (30 mL min^–1^), and the concentration of H_2_ and D_2_ was monitored with a mass spectrometer (BELMass, MicrotracBEL, Japan). Fourier transform infrared (FT-IR) spectra were measured using a spectrometer (FT/IR-6100, Jasco) equipped with a mercury–cadmium–tellurium detector at a resolution of 4 cm^–1^. Samples were pressed into self-supported disks. A disk was placed in a sealed and Ar-filled silica-glass cell equipped with NaCl windows to a closed gas-circulation system. The disk was heated under vacuum at 200 °C for 90 min. After the pretreatment, the disk was cooled to 25 °C under vacuum to obtain a background spectrum from the spectra of the N_2_-adsorbed samples. Pure N_2_ (99.99995%) was introduced into the system through a liquid nitrogen trap. X-ray photoelectron spectroscopy (XPS; ESCA-3200, Shimadzu, Mg Kα, 8 kV, 30 mA) was performed in conjunction with an Ar-filled glovebox, where the samples were moved to the ultra-high vacuum XPS apparatus through the Ar-filled glovebox without exposure to the ambient air. The binding energy was corrected with respect to the Au 4f_7/2_ peak of Au-deposited samples.

## 3 Results and Discussion

### 3.1 Iron Particles Loaded with an Electron-Donating Material

**Figure 2:**
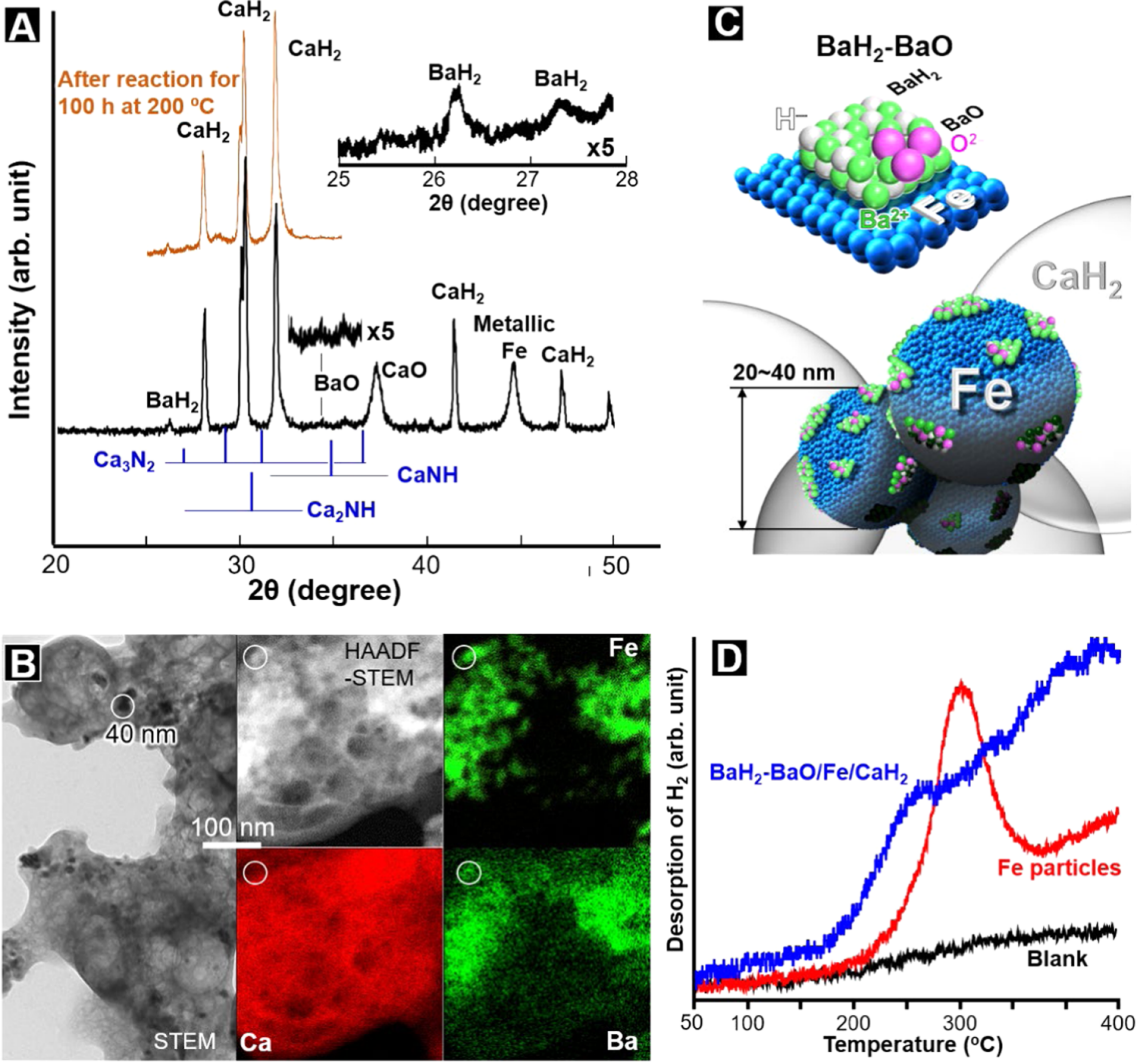
(A) Powder X-ray diffraction (XRD) patterns for BaH_2_-BaO/Fe/CaH_2_ after preparation and ammonia synthesis reaction for 100 h at 200 °C (see below). (B) High-angle annular dark-field scanning transmission electron microscopy (HAADF-STEM) and energy dispersive X-ray spectroscopy (EDX) images for BaH_2_-BaO/Fe/CaH_2_. (C) Schematic structure of BaH_2_-BaO/Fe/CaH_2_. (D) H_2_ (*m*/*z* = 2)-TPD profiles for metallic iron particles with and without BaH_2_-BaO (BaH_2_-BaO/Fe/CaH_2_ and Fe). Fe particles, metallic iron particles without BaO-BaH_2_, were obtained by heating 0.019 g of α-Fe_2_O_3_, with 0.081 g of CaH_2_ in a flow of H_2_ (45 mL min^–1^) at 300 °C for 2 h. After ammonia synthesis at 300 °C for over 20 h, the catalyst was cooled down to room temperature in a flow of Ar and was then heated at a rate of 1 °C min^–1^. Desorbed H_2_ was detected with a quadrupole mass spectrometer.

**Figure 3:**
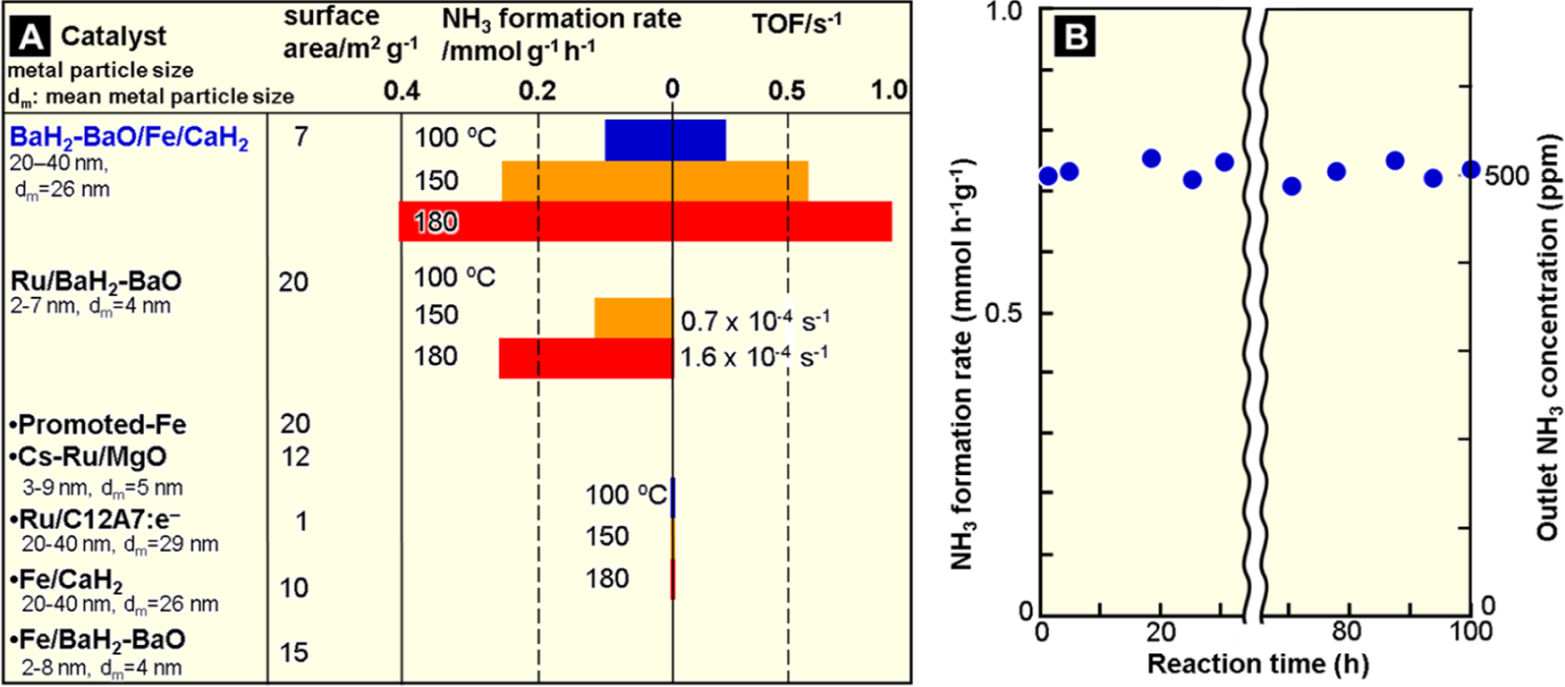
(A) Catalytic performance (ammonia formation rate and TOF) of the tested catalysts for ammonia synthesis at low temperatures. WHSV: 36 000 mL g^–1^ h^–1^, 0.9 MPa. (B) Time course of the ammonia formation rate on BaH_2_-BaO/Fe/CaH_2_ at 200 °C.

In several tested catalyst designs, metallic iron particles loaded with a mixture of BaO and BaH_2_ (BaH_2_-BaO/Fe/CaH_2_) were effective for low-temperature ammonia synthesis. Conventional supported iron catalysts, where iron nanoparticles are deposited on support particles with an electron-donating capability, cannot act as catalysts for ammonia synthesis below 200 °C. For example, an Fe nanoparticle (∼4 nm)-deposited BaH_2_-BaO mixture (Fe/BaH_2_-BaO) did not work for ammonia synthesis, even at 300 °C, although a Ru nanoparticle-deposited BaH_2_-BaO mixture (Ru/BaH_2_-BaO; see Supporting Information 1.2), which is similar to Fe/BaH_2_-BaO,^15^ could synthesize ammonia at low temperatures (Table S1). BaH_2_-BaO/Fe/CaH_2_ was readily prepared by heating Ba(NO_3_)_2_-impregnated Fe_2_O_3_ particles (20–40 nm) with CaH_2_ particles at 300 °C in a flow of H_2_. Figure 2A shows powder XRD profiles for BaH_2_-BaO/Fe/CaH_2_ immediately after preparation and after the ammonia synthesis reaction for 100 h at 200 °C (see Figure 3B). The XRD profiles were measured in Ar without exposure of the samples to the atmosphere. Diffraction peaks due to CaCO_3_ (2θ = 25°), calcium nitride species (Ca_3_N_2_, CaNH, and Ca_2_NH), and barium nitride (2θ = 27°) did not appear in the XRD pattern for either sample. H_2_O and oxygen species adsorbed on these samples, and all catalyst precursors stored in the Ar-filled glovebox were below the detection limit of mass spectrometry. Small diffraction peaks that could be assigned to BaH_2_ (2θ = 26.3 and 27.3°, JCPDS01-086-1744) were observed in the XRD profiles, together with those for CaH_2_, CaO, and metallic iron. On the other hand, there were no clear diffraction peaks for BaO or iron oxide species such as FeO, Fe_3_O_4_, and Fe_2_O_3_.

The Ba 3d_5/2_ XPS spectrum for BaH_2_-BaO/Fe/CaH_2_ is shown in Figure S2. A Ba 3d_5/2_ peak appeared at 781 eV in the XPS spectrum. This peak is generally insensitive to the electronic states of Ba compounds, and the binding energy is below 780 eV for most Ba species, which includes metallic Ba and BaO, except for a few rare exceptions.^18,19^ One such exception is BaH_2_, for which the binding energy for the Ba 3d_5/2_ peak is 782.9 eV.^20^ The Ba 3d_5/2_ peak for BaH_2_-BaO/Fe/CaH_2_ is located at a larger binding energy than those for most Ba species; the binding energy is close to that (781.5 eV) for a BaH_2_-BaO mixture obtained by heating a mixture of BaO and CaH_2_.^15^ The Fe 2p XPS spectrum for BaH_2_-BaO/Fe/CaH_2_ (Figure S3) immediately after preparation showed that the iron particle surface consists of Fe^0^ (48%), Fe^2+^ (26%), and Fe^3+^ (26%). These XRD and XPS results indicate that BaH_2_-BaO/Fe/CaH_2_ is mainly composed of BaH_2_, metallic iron, CaH_2_, and CaO, and the surfaces of BaH_2_ and metallic iron are partially oxidized. The BaH_2_ content in the Ba species was estimated to be more than 90 mol% from XRD patterns for various physical mixtures of BaH_2_ and BaO particles.

The bright-field STEM image (Figure 2B) reveals dark particles (>10–30 nm) due to either or both iron and Ba species on CaH_2_. HAADF-STEM and EDX images indicated that Ba species are present together with iron particles on CaH_2_; there are BaH_2_-deposited metallic iron particles on CaH_2_ particles in BaH_2_-BaO/Fe/CaH_2_, as shown in Figure 2C. In the XPS spectrum of BaH_2_-BaO/Fe/CaH_2_, a Ca 2p peak appeared at 347.0 eV in between those of CaH_2_ (347.4 eV) and CaO (346.4 eV) (Figure S4). As a result, BaH_2_-deposited metallic iron particles come into contact with CaH_2_ particles through a partially oxidized CaH_2_ surface.

We have previously reported that Ru nanoparticles in contact with alkaline earth metal hydrides MH_2_, such as CaH_2_ and BaH_2_, act as an effective catalyst for ammonia synthesis.^12,15,21^ Ru nanoparticles deposited on MH_2_ abstract H atoms from the near-surface due to substantial interaction between the transition metals and H^–^, and the H atoms move to the metal nanoparticles and are desorbed as H_2_ molecules to leave electrons in the H^–^ vacancies of MH_2_ (MH_2_ → M^2+^H_(2–*x*)_^–^e_*x*_^–^ + *x*H).^21^ The resultant M^2+^H_(2–*x*)_^–^e_*x*_^–^ behaves as a stable surface electride with a small work function comparable to those of metallic Li, Na, and K.^12,21^ The strong electron donation from M^2+^H_(2–*x*)_^–^e_*x*_^–^ to the transition metal nanoparticles enhances the cleavage of N_2_ molecules, which leads to high catalytic performance for ammonia synthesis. For this reason, the temperature at which H_2_ begins to desorb corresponds to that at which NH_3_ begins to desorb from the catalyst.^12,15^ This was confirmed on Ca^2+^H_2_^–^ (Ru/CaH_2_), a Ba^2+^H_2_^–^-BaO mixture (Ru/BaH_2_-BaO), and Ca^2+^F^–^H^–^ (Ru/CaFH) loaded with Ru nanoparticles.^12,15^ A large increase in H adatoms on the metal surface results in the spillover of H adatoms to electrons in the H^–^ vacancies of MH_2_, which forms H^–^ anions (M^2+^H_(2–*x*)_^–^e_*x*_^–^ + *x*H → MH_2_).^7^ H_2_-TPD measurements revealed that BaH_2_-BaO/Fe/CaH_2_ desorbs H_2_ above about 100 °C (Figure 2D), which implies that BaH_2_-BaO/Fe/CaH_2_ has strong electron-donating power at ≥100 °C. On the other hand, H_2_ was desorbed above 200 °C from Fe/CaH_2_ with metallic iron particles and without BaH_2_. The H_2_ desorption was due to H^–^ in CaH_2_ (see Supporting Information 1.3), and no ammonia formation was observed over metallic iron particle-deposited CaH_2_ (Fe/CaH_2_) at 200 °C (see Figure 3A). The XRD profile for Fe/CaH_2_ contained peaks due to CaH_2_, CaO, and metallic iron and coincided with that for BaH_2_-BaO/Fe/CaH_2_, except that the former lacked diffraction peaks due to BaH_2_. From these results, H_2_ desorption from BaH_2_-BaO/Fe/CaH_2_ below 200 °C can be attributed to BaH_2_ or/and CaH_2_ in the presence of BaH_2._ The work function for the (001) surfaces of Ba hydride species with hydride defects (Ba^2+^H_(2–1/9)_^–^e_1/9_^–^) was calculated to be 2.6 eV by density functional theory (DFT) (Figure S5). This value is smaller than that (Φ = 3.3 eV) for Ca hydride species with hydride defects (Ca^2+^H_(2–1/9)_^–^e_1/9_^–^),^15^ comparable to those for metallic K and Na (Φ = 2.3 and 2.4 eV, respectively). Based on the work function, H_2_ release from BaH_2_, followed by the formation of Ba hydride species with hydride defects, is more effective for strong electron-donating power.

### 3.2 Catalytic Performance of BaH_2_-BaO/Fe/CaH_2_ for Ammonia Synthesis

Figure 3A shows the catalytic performance for ammonia synthesis (100–200 °C, 0.9 MPa) over BaH_2_-BaO/Fe/CaH_2_ and Ru/BaH_2_-BaO. In Ru/BaH_2_-BaO, Ru metal nanoparticles (2–7 nm) are deposited on BaH_2_-BaO phase formed on CaH_2_ large particles (several 30 μm).^15^ The results for a commercial promoted iron catalyst (promoted-Fe) that consists of Fe, K_2_O, Al_2_O_3_, and CaO, MgO loaded with Ru nanoparticles and Cs oxide species (Cs-Ru/MgO), and Ru nanoparticles deposited on [Ca_24_Al_28_O_64_]^4+^(e^–^)_4_ (Ru/C12A7:e^–^) as benchmark catalysts are also shown. Table S1 summarizes the ammonia synthesis activities (300 °C, 0.9 MPa) of the tested materials and representative catalysts with physicochemical information, including metal particle sizes and surface areas. The promoted-Fe catalyst, which was first found by Haber, Bosch, and Mittasch more than 100 years ago and has been improved since then, is not inferior to most of the recently reported catalysts; only a handful of recent catalysts surpass the iron catalyst in terms of ammonia synthesis activity (Table S1).^8,15,22,23^ Nevertheless, the rate of ammonia formation for promoted-Fe below 300 °C was lower than that estimated by an Arrhenius equation, and the difference increased with decreasing temperature.^12^ Finally, promoted-Fe could not form ammonia at 200 °C. Cs-Ru/MgO and Ru/C12A7:e^–^ also did not work for ammonia synthesis below 200 °C. In contrast, BaH_2_-BaO/Fe/CaH_2_ revealed that iron can catalyze ammonia synthesis even at 100 °C. The rate of ammonia formation increased with the reaction temperature. Ammonia formation proceeded over BaH_2_-BaO/Fe/CaH_2_ without a significant decrease in activity for over 100 h (Figure 3B). The XRD profile for the catalyst after the long-term reaction is also shown in Figure 2A. There was no large difference in the XRD pattern between the catalyst after preparation and that after reaction for 100 h, which means that the catalyst structure, including CaH_2_ and BaH_2_, is stable during the reaction. The Fe 2p XPS spectrum for BaH_2_-BaO/Fe/CaH_2_ after the reaction showed that the iron particle surfaces consist of Fe^0^ (53%), Fe^2+^ (30%), and Fe^3+^ (17%); the Fe^0^:/Fe^2+^/Fe^3+^ atomic ratio on the iron surfaces after the reaction was similar to that (Fe^0^/Fe^2+^/Fe^3+^ = 48:26:26) for the catalyst immediately after preparation. Taking into account the number of zero-valent Fe atoms on the iron surface of the catalyst, the reaction for 100 h was estimated to give a turnover number of ca. 600 thousand (6.12 × 10^5^); BaH_2_-BaO/Fe/CaH_2_ with a new type of catalyst structure acts as a stable catalyst. Table 1 summarizes the outlet ammonia concentrations and ammonia spacetime yields for the BaH_2_-BaO/Fe/CaH_2_ and commercial promoted-Fe catalysts, in addition to the theoretical outlet ammonia concentrations at 100, 200, and 300 °C. The outlet ammonia concentrations for both iron catalysts were considerably smaller than the equilibrium ammonia concentration. While the ammonia concentration for the former catalyst increased in proportion to the catalyst weight, an increase in catalyst weight did not lead to ammonia detection over the latter commercial iron catalyst below 200 °C.

**Table 1:** Outlet Ammonia Concentrations and Spacetime Yields for BaH_2_-BaO/Fe/CaH_2_ and Commercial Promoted-Fe (0.9 MPa, H_2_/N_2_ = 3:1, WHSV: 36 000 mL g cat^–1^ h^–1^)

catalyst	temperature/°C	outlet NH_3_ conc. (%) (STY/kgNH_3_ kg-cat^–1^ h^–1^)^a^	theoretical outlet NH_3_ conc. (%) (STY/kgNH_3_ kg-cat^–1^ h^–1^)^a^
BaFL-BaO/Fe/CaH_2_	300	0.353 (0.86)	14.5 (35.2)
	200	0.050 (0.12)	48.6 (118.0)
	100	0.007 (0.02)	93.0 (225.8)
commercial promoted-Fe^b^	300	0.383 (0.93)	14.5 (35.2)
	200	—	48.6 (118.0)
	100	—	93.0 (225.8)

a Spacetime yield.

b Outlet NH_3_ concentration of commercial promoted-Fe catalyst reaches 28% at 450 °C under 10 MPa (STY: 755.5 kgNH_3_ kg-cat^–1^ h^–1^). This is consistent with the theoretical NH_3_ concentration.

Next, CaH_2_, BaH_2_, Fe/CaH_2_, and BaH_2_-BaO/Fe/CaH_2_ were examined under ammonia synthesis conditions (0.9 MPa, 200 °C). CaH_2_ and BaH_2_ did not synthesize ammonia at 200 °C, as shown in Table 2. XRD analysis indicated that nitrogen-containing species such as metal nitrides, imides, and amides were not formed on any of the samples after the experiment. Therefore, these alkaline earth metal hydrides themselves cannot react with N_2_ and are not effective for ammonia synthesis from H_2_ and N_2_ under the present reaction conditions. In ammonia synthesis catalytic systems where alkali metal and alkaline earth metal hydrides directly form ammonia, XRD diffraction peaks due to these metal nitride species are observed on the catalysts after reaction because ammonia has a high reactivity with alkali metal and alkaline earth metal hydrides, and ammonia formation on the metal hydride surfaces readily forms metal nitrides or imides.^9,10^ On the other hand, nitrogen-containing alkaline earth metal species were not detected on BaH_2_-BaO/Fe/CaH_2_, even after an ammonia synthesis reaction for 100 h, as described in Section 3.1. No formation of nitrogen-containing alkaline earth metal species on BaH_2_-BaO/Fe/CaH_2_, CaH_2_, and BaH_2_ under ammonia synthesis conditions and the lack of reactivity of alkaline earth metal hydrides with N_2_ imply that ammonia formation, *i.e.*, N_2_ cleavage and the formation of NH*_n_* species do not proceed on alkaline earth metal hydrides in BaH_2_-BaO/Fe/CaH_2_; metallic iron on the catalyst directly synthesizes ammonia, as in the case of conventional transition metal catalysts. H_2_ desorption from low temperature on BaH_2_-BaO/Fe/CaH_2_ (Figure 2D) indicates the formation of hydride defect-containing alkaline earth metal hydride species with strong electron-donating capability that can facilitate N_2_ dissociative adsorption on transition metal surfaces. The strong electron donation by such electron-donating species due to BaH_2_ or/and CaH_2_ can enhance the catalytic activity of metallic iron.

**Table 2:** Ammonia Formation Rates for the Tested Samples (0.9 MPa, 200 °C, WHSV: 36 000 mL g^–1^ h^–1^, N_2_/H_2_ = 1:3)

sample	CaH_2_	BaH_2_	Fe/CaH_2_	BaH_2_-BaO/Fe/CaH_2_
ammonia formation rate/μmol g^–1^ h^–1^	—	—	—	720

Although transition metal particles on alkali metal and alkaline earth metal hydrides such as Co/LiH and Ru/CaH_2_ have been reported to exhibit higher catalytic performance for ammonia synthesis than transition metal particles on metal oxides,^9,21^ Fe/CaH_2_ did not form ammonia at the reaction temperature (Table 2). There is no substantial difference in the structures of BaH_2_-BaO/Fe/CaH_2_ and Fe/CaH_2_, except that BaH_2_ is not deposited on the metallic iron particles in the latter. From these results, the catalytic activity of metallic iron on BaH_2_-BaO/Fe/CaH_2_ is expected to be enhanced by BaH_2_ or/and CaH_2_ in the presence of BaH_2_. In the latter case, CaH_2_ acts as not only a support for metallic iron particles but also an electron-donating material and is correlated with metallic iron through BaH_2_. We cannot distinguish between the two possible mechanisms because we are not able to prepare only BaH_2_-deposited metallic iron particles without using CaH_2_ at present.

**Figure 4:**
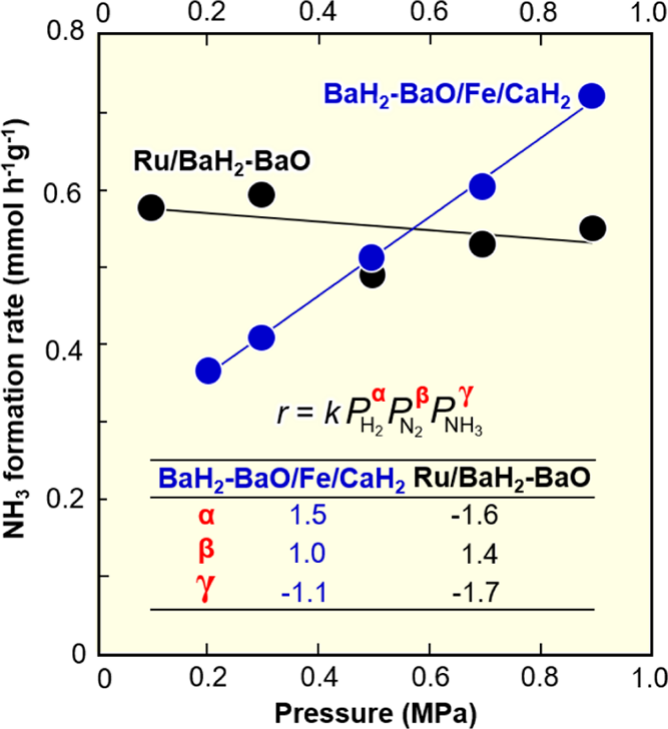
Correlation of the ammonia formation rate with pressure and the reaction orders for H_2_, N_2_, and NH_3_ over BaH_2_-BaO/Fe/CaH_2_ and Ru/BaH_2_-BaO at 200 °C.

**Figure 5:**
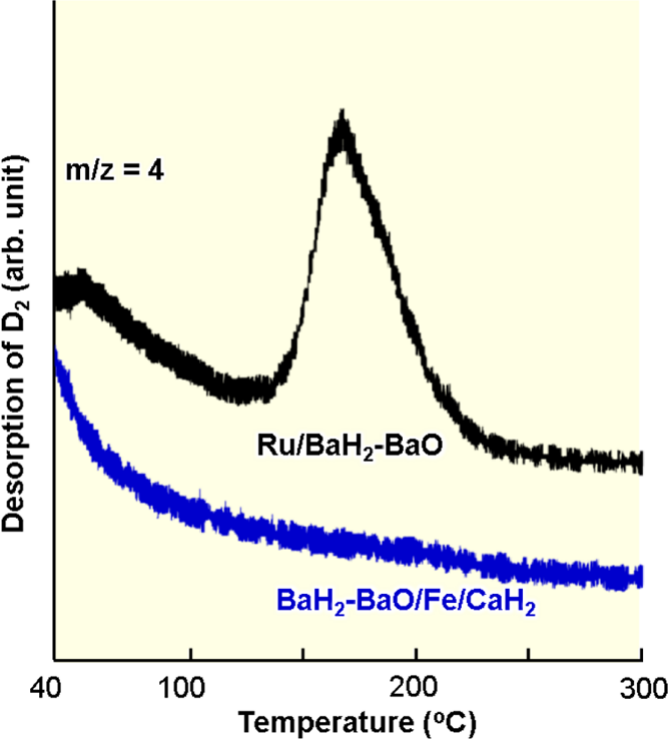
D_2_-TPD (1 °C min^–1^) for BaH_2_-BaO/Fe/CaH_2_ and Ru/BaH_2_-BaO. The catalysts were heated at 300 °C in a flow of Ar to remove H adatoms from the Fe and Ru surfaces. After they were cooled down to 25 °C, D adatoms were adsorbed onto the Fe and Ru surfaces in a flow of D_2_ (15 mL min^–1^, 30 min) at 25 °C.

A notable feature in Figure 3A is the catalytic activity and efficiency for ammonia formation of Ru/BaH_2_-BaO. Ru/BaH_2_-BaO above 300 °C is of the highest standard, as shown in Table S1.^15^ However, Ru/BaH_2_-BaO could not synthesize ammonia at 100 °C, and the ammonia formation rate was inferior to BaH_2_-BaO/Fe/CaH_2_ below 200 °C, although both catalysts use the same electron-donating material. The difference is further emphasized with respect to the ammonia formation turnover for the surface zero-valent Fe and Ru atoms (turnover frequency; TOF). In Figure 3A, BaH_2_-BaO/Fe/CaH_2_ exhibits TOFs that are several thousand times higher than Ru/BaH_2_-BaO. In heterogeneous catalysts that adopt Ru, Co, and Ni as the active sites for ammonia synthesis, the maximum TOF is at most less than 0.17 s^–1^, even at 400 °C (Table S2).^11^ However, the TOF of the iron catalyst was 0.23 s^–1^ at 100 °C and reached 12.3 s^–1^ at 300 °C. To understand the difference in catalysis between Fe and Ru promoted by the same electron-donating material, the correlation of ammonia formation rate with total pressure and the reaction orders in the rate equation on BaH_2_-BaO/Fe/CaH_2_ and Ru/BaH_2_-BaO were measured at 200 °C and are summarized in Figure 4. The ammonia formation rate for Ru/BaH_2_-BaO did not increase with increasing pressure, and the reaction order for H_2_ showed a negative value of −1.6; an increase in reactant concentration cannot lead to an increase in product formation. These results are inconsistent with kinetics theory and are clearly due to hydrogen-poisoning,^13,14^ where the Ru surface on Ru/BaH_2_-BaO is severely poisoned by H adatoms and cannot exhibit satisfactory catalytic performance for ammonia formation. Such a hydrogen-poisoned Ru surface would not show a high TOF. On the other hand, the rate of ammonia formation increased in proportion to the pressure over BaH_2_-BaO/Fe/CaH_2_ to show a positive value of +1.5 as the reaction order for H_2_. As a result, the iron catalyst is not strongly affected by hydrogen-poisoning, even at 200 °C, which results in a much higher TOF than those of Ru-based catalysts. One possible explanation for hydrogen-poisoning due to the H_2_ adsorption/desorption equilibrium on transition metals is the density and strength of the bonds between H adatoms and surface transition metal atoms. A larger amount of H atoms is adsorbed on transition metal surfaces with decreasing temperature; therefore, we may clarify the effect of hydrogen-poisoning by observing H_2_ desorption from BaH_2_-BaO/Fe/CaH_2_ and Ru/BaH_2_-BaO at low temperatures. Figure 5 shows molecular deuterium (D_2_)-TPD measurements for both the catalysts. The desorption of D adatoms due to gas-phase D_2_ can be distinguished from the desorption of H adatoms originated from H^–^ anions in BaH_2_ through transition metals by the use of D_2_-TPD. Most D adatoms are desorbed as D_2_ from the iron catalyst at 40–100 °C (see Supporting Information 1.4). It was confirmed that D_2_ desorption from the iron catalyst is consistent with H_2_ desorption from a single-crystal iron surface.^24^ This suggests that the iron surface provides sufficient adsorption sites for N_2_ and N adatoms without a strong influence of hydrogen-poisoning under ammonia synthesis conditions above 100 °C. In the case of Ru/BaH_2_-BaO, a large D_2_ desorption peak was observed at 125–225 °C; Ru binds to H adatoms more tightly than iron, and these H adatoms can cause hydrogen-poisoning on Ru over a broad temperature range. Co and Ni surfaces have also been found to strongly adsorb H adatoms that are desorbed as H_2_ at >150–200 °C as with Ru.^25−27^ These results imply that many transition metals used to synthesize ammonia can be significantly affected by hydrogen-poisoning at low temperatures, and iron is an exceptional transition metal that prevents hydrogen-poisoning. The difference in hydrogen-poisoning may be expressed as the difference in TOF among BaH_2_-BaO/Fe/CaH_2_ and the other transition metal-based catalysts.

### 3.3 Reaction Mechanism of Ammonia Formation over BaH_2_-BaO/Fe/CaH_2_

**Figure 6:**
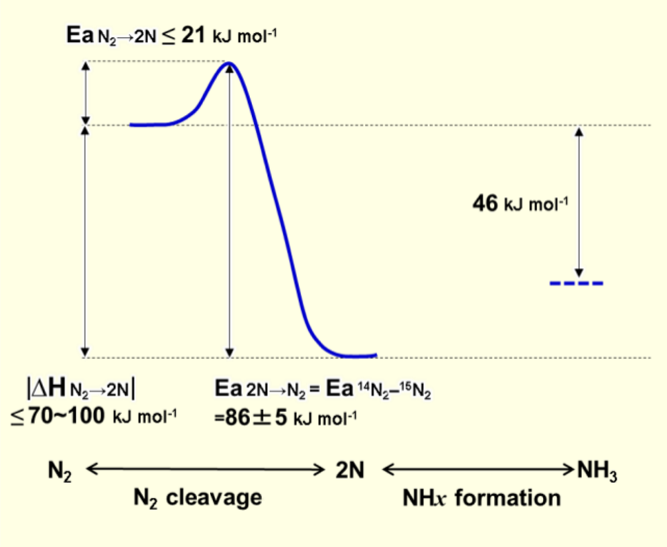
Potential energy diagram for dissociative N_2_ adsorption on BaH_2_-BaO/Fe/CaH_2_.

Ammonia synthesis over the iron catalyst was further studied to understand the reaction mechanism. Figure 6 is an energy diagram estimated from the heat of dissociative adsorption of N_2_ (Δ*H* N_2_ → 2N) and the activation energy for N_2_ desorption from N adatoms (Ea 2N → N_2_) on BaH_2_-BaO/Fe/CaH_2_. Figures S6 and S7 are Arrhenius plots for ammonia synthesis and the ^14^N_2_-^15^N_2_ isotropic exchange reaction (^14^N_2_ + ^15^N_2_ ↔ 2^14^N^15^N) over BaH_2_-BaO/Fe/CaH_2_, respectively. From Figure S7, the ^14^N_2_-^15^N_2_ isotropic exchange reaction (Ea ^14^N_2_-^15^N_2_) was estimated to be 86 ± 5 kJ mol^–1^. It has been confirmed that the apparent activation energy for the N_2_ isotopic exchange reaction is equal to the activation energy for N_2_ desorption (Ea 2N → N_2_) by the recombination of N adatoms on ammonia synthesis heterogeneous catalysts.^28−30^ Therefore, the rate-determining step for the N_2_ isotopic exchange reaction is the same as the activation energy for the recombination of N adatoms (Ea ^14^N_2_–^15^N_2_ = Ea 2N → N_2_ = 86 ± 5 (81–91) kJ mol^–1^). The dissociative adsorption heat for N_2_ into N adatoms (Δ*H* N_2_ → 2N) on a pure iron surface has been estimated to be −70 to −100 kJ mol^–1^.^31^ Electron donation from BaH_2_ to N adatoms stabilizes these atoms, which results in larger dissociative adsorption heat on BaH_2_-BaO/Fe/CaH_2_ than that on pure Fe.^28,32^ The dissociative adsorption heat of N_2_ on the iron surface of BaH_2_-BaO/Fe/CaH_2_ (Δ*H* N_2_ → 2N) would therefore be less than −70 to −100 kJ mol^–1^ (|Δ*H* N_2_ → 2N| ≥ 70–100 kJ mol^–1^). From these results, the activation energy for N_2_ cleavage (Ea N_2_ → 2N) on BaH_2_-BaO/Fe/CaH_2_ is estimated to be below 21 kJ mol^–1^ from Ea 2N → N_2_ and Δ*H* N_2_ → 2N (Ea N_2_ → 2N = Ea 2N → N_2_**–** |Δ*H* N_2_ → 2N|). The apparent activation energy (Ea NH_3_) for ammonia synthesis over BaH_2_-BaO/Fe/CaH_2_ was estimated to be 40 ± 5 kJ mol^–1^ from Figure S6. Although BaH_2_-BaO/Fe/CaH_2_ can therefore be categorized as an ammonia synthesis catalyst with a small apparent activation energy, as shown in Table S2, the apparent activation energy does not directly reflect the activation energy for N_2_ cleavage (Ea N_2_ → 2N).

Next, we examined the isotope effect over the iron catalyst through ^15^NH_3_ and ^14^NH_3_ formation from ^15^N_2_-H_2_ and ^14^N_2_-H_2_, respectively. The ratio of the dissociation probability for ^15^N_2_ to that for ^14^N_2_ on transition metal surfaces has been reported to be 0.72–0.79.^33^ The results for ^15^NH_3_ and ^14^NH_3_ formation over BaH_2_-BaO/Fe/CaH_2_ in respective flows of ^15^N_2_-H_2_ and ^14^N_2_-H_2_ are summarized in Table 3. There was no significant difference in the ammonia formation rates between ^15^N_2_-H_2_ and ^14^N_2_-H_2_ over the iron catalyst at 200–300 °C. Many years of research, including microkinetics, iron single-crystal surfaces, and isotope experiments, indicate that N_2_ dissociation is the dominant process for ammonia synthesis over iron catalysts.^34,35^ On the other hand, the results in Table 3 suggest that ammonia synthesis over BaH_2_-BaO/Fe/CaH_2_ cannot be simply explained only by the reaction mechanism for conventional iron catalysts.

**Table 3:** Formation Rates of ^15^NH_3_ and ^14^NH_3_ over BaH_2_-BaO/Fe/CaH_2_ in a Flow of ^15^N_2_-H_2_ and ^14^N_2_-H_2_, Respectively (0.1 MPa, WHSV: 36 000 mL g^–1^ h^–1^, N_2_/H_2_ = 1:3)

	NH_3_ formation rate/mmol g^–1^ h^–1^		r^15^NH_3_
temperature/°C	r^15^NH_3_	r^14^NH_3_	r^14^NH_3_
200	0.35	0.38	0.92
250	0.59	0.58	1.02
300	1.25	1.28	0.98

**Figure 7:**
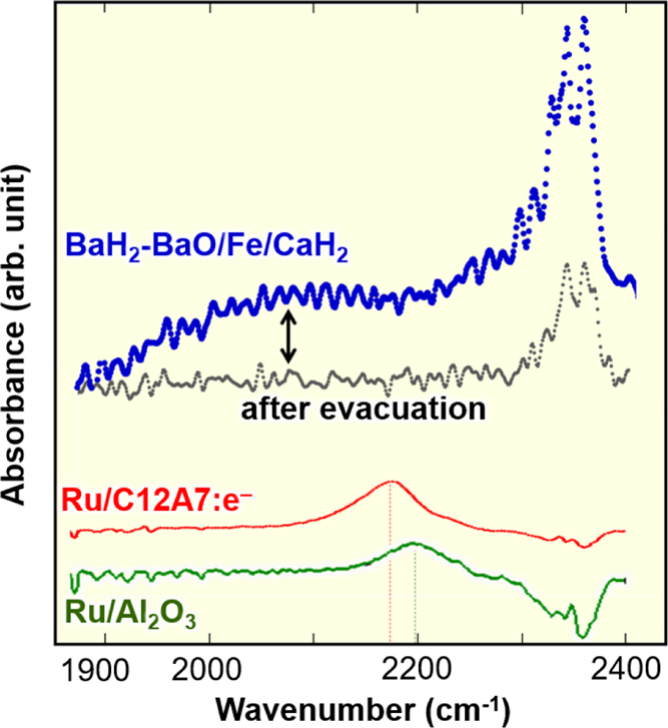
FT-IR spectra for N_2_-adsorbed BaH_2_-BaO/Fe/CaH_2_, Ru/C12A7:e^–^, and Ru/Al_2_O_3_. 10 kPa of N_2_ at 25 °C.

These results may indicate facile N_2_ cleavage on BaH_2_-BaO/Fe/CaH_2_ by weakening of the strong N≡N triple bonds. This requires electron donation from electron-donating materials to the antibonding π* orbitals of the adsorbed N_2_ via transition metal d-orbitals (*i.e.*, back-donation) to be boosted. The strong back-donation would weaken the N≡N stretching vibration by elongation of the N≡N bond. In this study, Fourier transform infrared (FT-IR) spectroscopy measurements using ^14^N_2_ and ^15^N_2_ as probe molecules were used to observe the N≡N stretching of N_2_ adsorbed on transition metal surfaces. In the FT-IR spectrum for ^14^N_2_ adsorbed on Ru nanoparticles deposited on Al_2_O_3_ (Ru/Al_2_O_3_) (Figure 7), the N≡N stretching band (νN_2_) appeared at 2150–2250 cm^–1^ (peak top: 2200 cm^–1^), which is much lower than that of gaseous N_2_ (2744 cm^–1^). The large red shift indicates that the electron donation from Ru to the antibonding π* orbitals of adsorbed N_2_ elongates the N≡N bond. The νN_2_ band was further red-shifted to 2100–2200 cm^–1^ (peak top: 2175 cm^–1^) in the spectrum for N_2_ adsorbed on Ru/C12A7:e^–^, where the energy barriers for N–H species formation are larger than that of N_2_ cleavage. The stronger electron donation from C12A7:e^–^ to N_2_ molecules adsorbed on Ru facilitates the generation of N adatoms and reduces the activation energy for the latter below those for the former.^28^ The FT-IR spectrum for ^14^N_2_-adsorbed BaH_2_-BaO/Fe/CaH_2_ in Figure 7 shows that a broad band is observed in the range of 2000–2175 cm^–1^, which is lower than that of Ru/C12A7:e^–^, and disappears after the removal of N adatoms by evacuation. The broad band was assigned to νN_2_ by the ^15^N_2_ adsorption experiment (Figure S8). Thus, the iron catalyst is more electron-donating than Ru/C12A7:e^–^ and can split adsorbed N_2_ molecules more easily than the latter. H_2_ desorption from low temperatures on BaH_2_-BaO/Fe/CaH_2_ (Figure 2D) indicates the formation of hydride defect-containing alkaline earth metal hydride species with strong electron-donating capability; the strong electron-donating power of the iron catalyst can be attributed to the formation of such hydride species.

**Figure 8:**
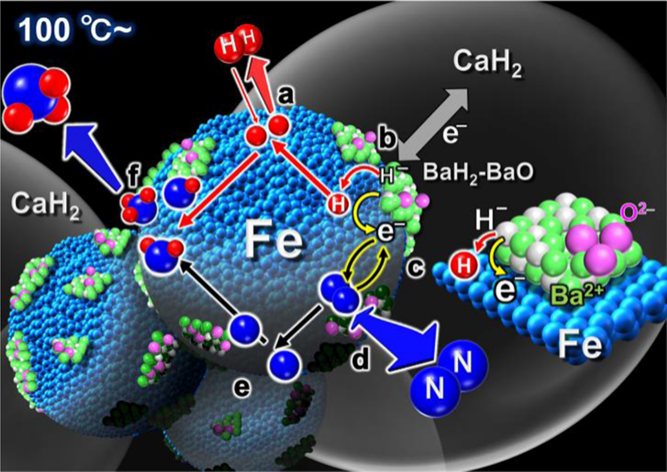
Schematic of the reaction mechanism for ammonia synthesis over BaH_2_-BaO/Fe/CaH_2_.

As a summary of these results, the mechanism postulated for ammonia synthesis over BaH_2_-BaO/Fe/CaH_2_ is shown schematically in Figure 8. Only BaH_2_-BaO/Fe/CaH_2_, BaH_2_-deposited metallic iron particles on CaH_2_ particles, catalyzed ammonia synthesis at low temperatures in the tested catalyst designs that adopted iron as the active sites. The catalyst designs based on conventional supported metal catalysts, including the iron nanoparticles-deposited BaH_2_-BaO mixture (Fe/BaH_2_-BaO), were not effective for low-temperature ammonia synthesis. The experimental results imply that ammonia formation, including N_2_ cleavage and the formation of NHn species, proceeds on the metallic iron surface of BaH_2_-BaO/Fe/CaH_2_ as with ammonia synthesis over conventional transition metal catalysts. Most H adatoms on the iron surfaces desorb as H_2_ below 100–150 °C (Figure 5) so that H_2_ adsorption/desorption equilibrium is shifted toward H_2_ desorption at low temperatures (Figure 8a). For this reason, the H adatom concentration is not so high over the entire reaction temperature range above 100–150 °C. The low H adatom concentration induces the alkaline earth metal hydride MH_2_ to exhibit strong electron-donating capability. The iron surface in contact with MH_2_ can abstract H atoms from MH_2_ and release them as H_2_ molecules (MH_2_ ↔ M^2+^H_(2–*x*)_^–^e_*x*_^–^ + *x*/2H_2_). This forms alkaline earth metal hydride species with electrons and hydride defects (M^2+^H_(2–*x*)_^–^e_*x*_^–^) that exhibit strong electron-donating capability. The strong electron donation to metallic iron is considered to be due to BaH_2_ or/and a combination of BaH_2_ and CaH_2_. In the latter case, electrons derived from CaH_2_ would be correlated with the metallic iron surface through BaH_2_ (Figure 8). In either case, electron donation from BaH_2_, as shown in Figure 8b,c, may play an important role in enhancing the catalytic activity of metallic iron. The shift of the H_2_ adsorption/desorption equilibrium toward H_2_ desorption on the iron surfaces would not only give the strong electron-donating capability to BaH_2_ but also provide sufficient adsorption sites for N_2_ molecules on the iron surface (Figure 8d). Electrons are transferred from the resulting alkaline earth metal hydride species with hydride defects to N_2_ molecules adsorbed on iron surfaces. FT-IR measurements (Figure 7) revealed that the antibonding π* orbitals of adsorbed N_2_ are significantly stimulated by the strong electron donation. The strong electron donation can facilitate the cleavage of adsorbed N_2_ to N adatoms (Figure 8d,e). The generated N adatoms react with H adatoms to form ammonia through the formation of N–Hn species (Figure 8f). These reactions on BaH_2_-BaO/Fe/CaH_2_ proceed at more than ca. 100 °C (Figure 3A) while hydrogen-poisoning is prevented. On the other hand, other transition metals such as Ru have tightly adsorbed H adatoms on their surfaces that cannot be easily removed as H_2_ below 150–200 °C. The H adatoms would reduce the number of the adsorption sites for N_2_ molecules and N adatoms and affect the reaction over the entire temperature range.

## 4 Conclusions

The present study demonstrates that metallic iron particles can catalyze ammonia synthesis from H_2_ and N_2_ at 100 °C in combination with BaH_2_. The iron catalyst exhibits a much higher TOF than heterogeneous catalysts with other transition metals as the reaction sites. This can be attributed to the intrinsic nature of the metallic iron surface to desorb H adatoms as H_2_ molecules at low temperatures of <100 °C. Many transition metals used to synthesize ammonia, such as Ru, Co, and Ni, bind to H adatoms more tightly than iron, and these H adatoms can cause hydrogen-poisoning on these transition metals over a broad temperature range >150–200 °C. As a result, iron is considered to be an exceptional transition metal that prevents hydrogen-poisoning. Strong electron donation to metallic iron particles in the presence of BaH_2_ also makes a significant contribution to low-temperature ammonia formation over the iron catalyst, which indicates that alkaline metal hydride can enhance the catalytic performance of iron. Therefore, iron, which was first found to catalyze ammonia formation at high temperatures by Mittasch over 100 years ago, may also be effective for low-temperature ammonia synthesis.

## Supporting Information Available

Additional discussion, XPS spectra, DFT calculation, and FT-IR spectra (PDF)
