# Supplementary material for: Low-Temperature Ammonia Synthesis on Iron Catalyst with an Electron Donor
Source: J Am Chem Soc. 2023 Mar 30;145(14):7888–97. doi: 10.1021/jacs.2c13015 (PMC10103163; doi:10.1021/jacs.2c13015)
Supplement: Supplementary file 1 — ja2c13015_si_001.pdf [file ja2c13015_si_001.pdf]

Supporting Information for

Low temperature ammonia synthesis on iron catalyst with an electron donor

Masashi Hattori, Natsuo Okuyama, Hiyori Kurosawa, Michikazu Hara*

Laboratory for Materials and Structures, Tokyo Institute of Technology, 4259 Nagatsuta, Midori-ku, Yokohama 226–8503, Japan

*E-mail: mhara@msl.titech.ac.jp

Contents

1. Additional information
   1.1. Reactor set-up
   Figure S1
   1.2. Ammonia synthesis over BaH_2-BaO/Ru/CaH_2
   1.3. H_2 desorption from BaH_2-BaO/Fe/CaH_2 and Fe/CaH_2
   1.4. D_2 desorption from BaH_2-BaO/Fe/CaH_2
2. Supplementary Figures and Tables
   Figure S2-S8, Tables S1 and 2
3. References

1. Additional information

1.1. Reactor set-up

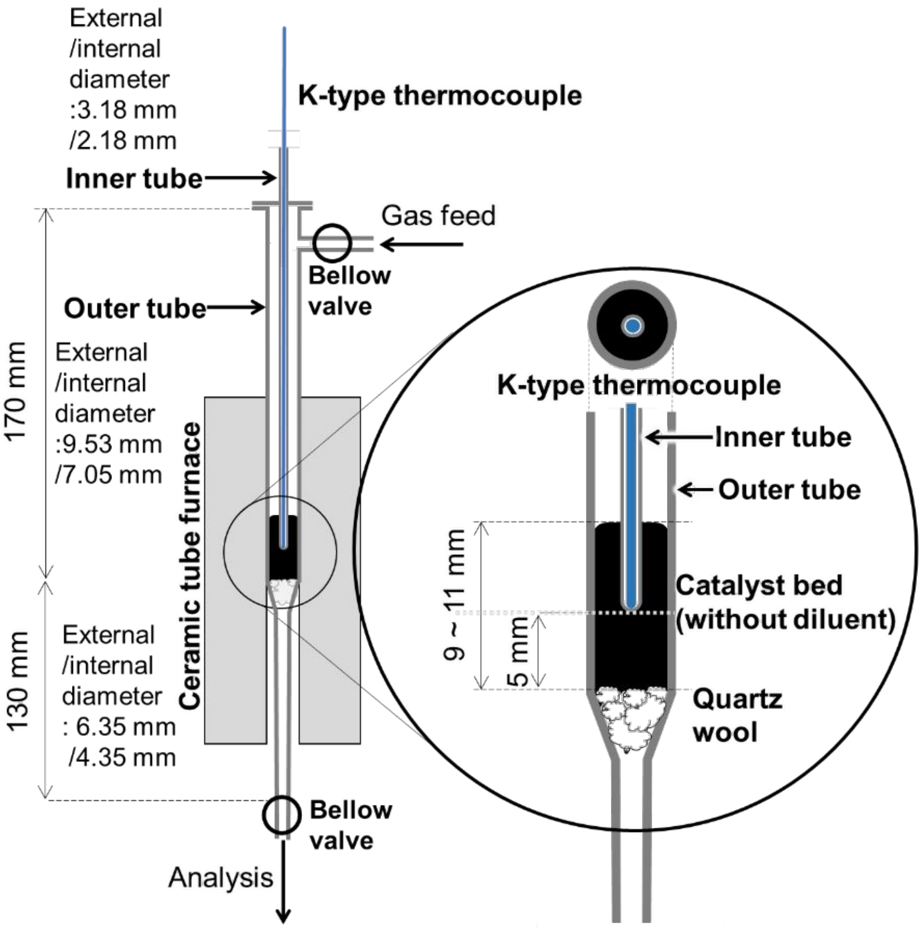

Figure S1. Schematic illustration of the reactor set-up.

The schematic illustration of the reactor set-up in this study is shown in Figure S1. The stainless steel fixed bed reactor (SUS 304) used for evaluation of the catalytic performance included a duplex tube with a 300 mm SUS 304 outer reaction tube (170 mm (external/internal diameter: 9.53 mm/7.05 mm) + 130 mm (external/internal diameter: 6.35 mm/4.35 mm)) and a SUS 304 inner tube (200 mm (external/internal diameter: 3.18 mm/2.18 mm)) as a thermocouple well. The front end of the inner tube was closed. Stainless steel bellow valves were connected to both ends of the outer tube. The middle of the outer reaction tube that was vertically located was filled with catalyst particles. The height of catalyst bed was 9~11 mm. In this study, the catalytic activities of all catalysts were examined without adding any diluent to catalyst bed. A sheathed K-type (chromel-alumel) thermocouple (1.8 mm in diameter) was inserted into the inner tube and the tip of the sheathed thermocouple was in contact with the closed front end of the inner tube which reached the middle of the

catalyst bed (a height of ca. 5 mm from the bottom of the catalyst bed) as shown in Figure S1; the reaction temperature in this study represents that of the middle of the catalyst bed. The reactor was heated in a 800 W ceramic tube furnace (Asahi Rika ARF1-200 (Kanthal wire)).

In preparation for alkaline earth metal hydride-containing catalysts, the reactor was loaded with a mixture of catalyst precursors in an Ar-filled glovebox, as shown in Figure S1. It was confirmed in a quartz reactor that there is no significant difference in catalyst bed height among the mixture of catalyst precursors, the catalyst immediately after preparation in a flow of H_2 and the catalyst after ammonia synthesis reaction. The reactor loaded with the mixture of catalyst precursors was removed from the Ar-filled glovebox to the atmosphere and connected to a flow reaction system without exposure of the mixture in the reactor to the atmosphere. The reactor was heated in the ceramic tube furnace in a flow of H_2, which resulted in the tested catalysts. The catalytic activities of the prepared catalysts were examined by flowing N_2-H_2 into the reactor.

1.2. Ammonia synthesis over BaH_2-BaO/Ru/CaH_2

BaH_2-BaO/Ru/CaH_2 was prepared from commercial metallic Ru nanoparticles (average particle size: 30 nm) in a similar manner to BaH_2-BaO/Fe/CaH_2. However, BaH_2-BaO/Ru/CaH_2 did not synthesize ammonia under the present reaction conditions. It was confirmed that an increase in the Ru particle size (from 4 nm to >10 nm) in the Ru/BaH_2-BaO catalyst by increasing the amount of Ru deposition significantly decreased the catalytic activity for ammonia synthesis, which suggests that BaH_2-BaO loading is not effective for large metallic Ru particles. The details are currently under investigation.

1.3. H_2 desorption from BaH_2-BaO/Fe/CaH_2 and Fe/CaH_2

Prior to H_2-TPD experiments, the sample after the ammonia synthesis experiment at 300 °C for over 20 h was kept at 300 °C for 1 h in a flow of Ar and then cooled down to room temperature. The sample was then heated at a rate of 1 °C min^{-1} in an Ar flow (see the figure caption Figure 2D). NH_3- and N_2-TPD measurements confirmed that ammonia and its derivatives are not adsorbed on the sample before H_2-TPD experiment. Hydrogen adatoms on the metallic iron-alkaline earth metal hydride system are desorbed as hydrogen molecules below about 100 °C, as shown in Figure 5; therefore, it is difficult for the catalytic system to adsorb hydrogen before H_2-TPD experiment under the present experimental conditions. In addition, the H_2 and H_2O concentrations in the TPD experimental system were below the detection limit of mass spectrometry. Therefore, it was considered that H_2 desorption observed on Fe/CaH_2 is due to hydride anions in CaH_2.

1.4. D_2 desorption from BaH_2-BaO/Fe/CaH_2

There are two types of H_2 desorption on transition metal-alkaline earth metal hydride systems; recombination among H adatoms from gas phase H_2 and H_2 derived from H^- anions in alkaline earth metal hydrides. The H_2 desorption temperature for the latter is higher than that for the former because it proceeds through a multi-step process, including hydride defect formation, migration of hydrogen to transition metal surfaces and recombination of hydrogen. In the case of BaH_2-BaO/Fe/CaH_2, there is a large difference (about 100 °C) between the

former and latter desorption temperatures. It was confirmed that H_2 is desorbed from the catalyst at >100 °C after D_2 desorption below 100 °C. HD formation was not observed on BaH_2-BaO/Fe/CaH_2, because almost all the D adatoms are desorbed as D_2 before the migration of H in the hydride to the Fe surface.

2. Supplementary Figures and Tables

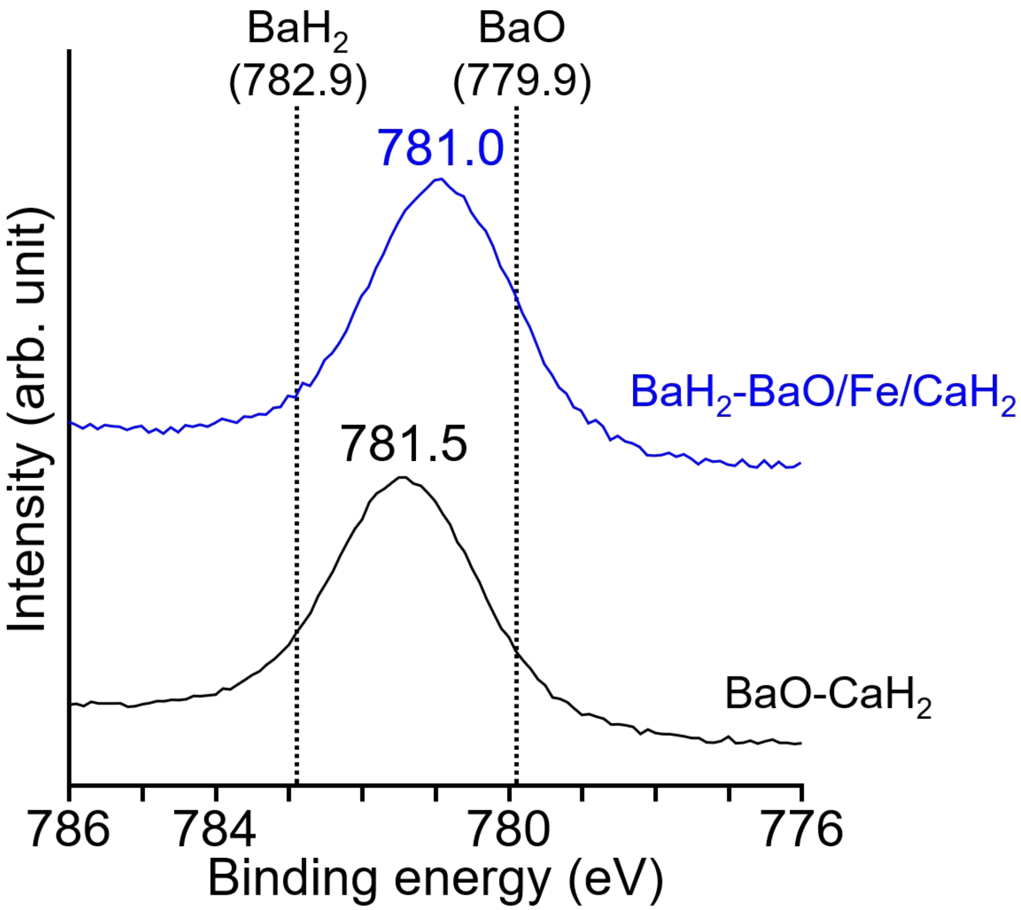

Figure S2. Ba $3d_{5/2}$ XPS spectra[1,2] for BaH_2-BaO/Fe/CaH_2 and BaH_2-BaO mixture. BaH_2-BaO mixture was prepared by heating a mixture of BaO and CaH_2.[3]

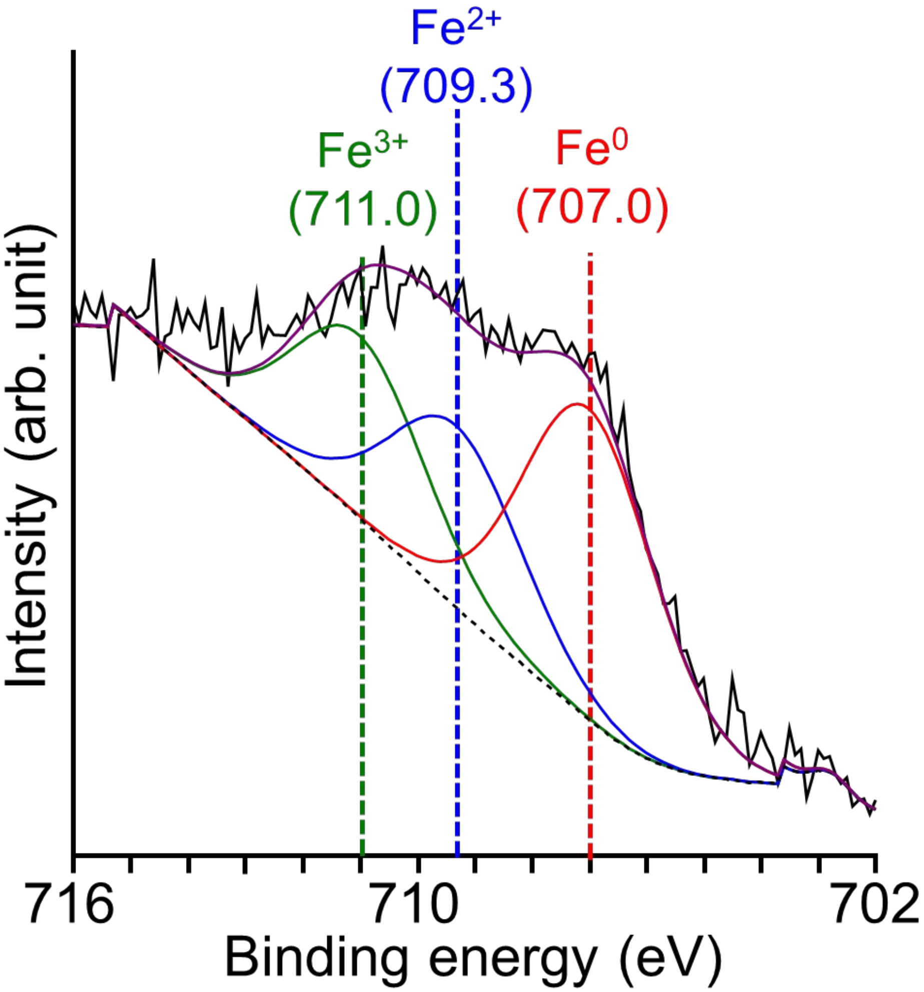

Figure S3. Fe $2p_{3/2}$ XPS spectrum[4] for BaH_2-BaO/Fe/CaH_2.

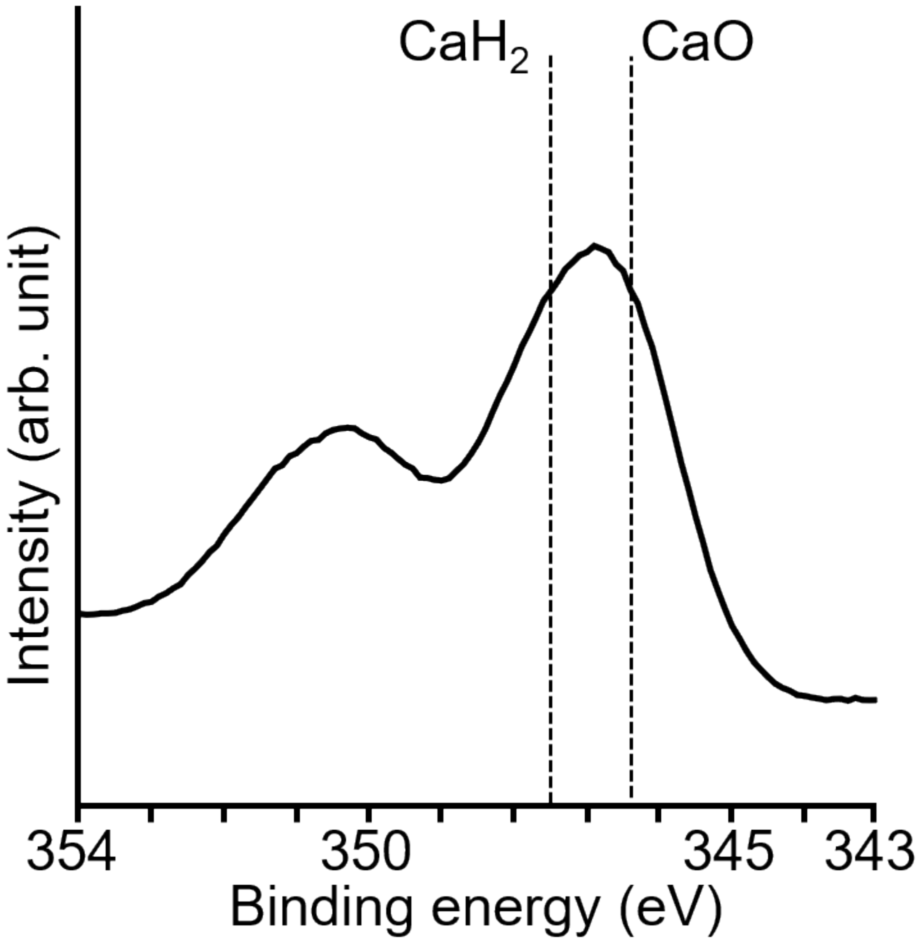

Figure S4. Ca 2p XPS spectrum for BaH_2-BaO/Fe/CaH_2.

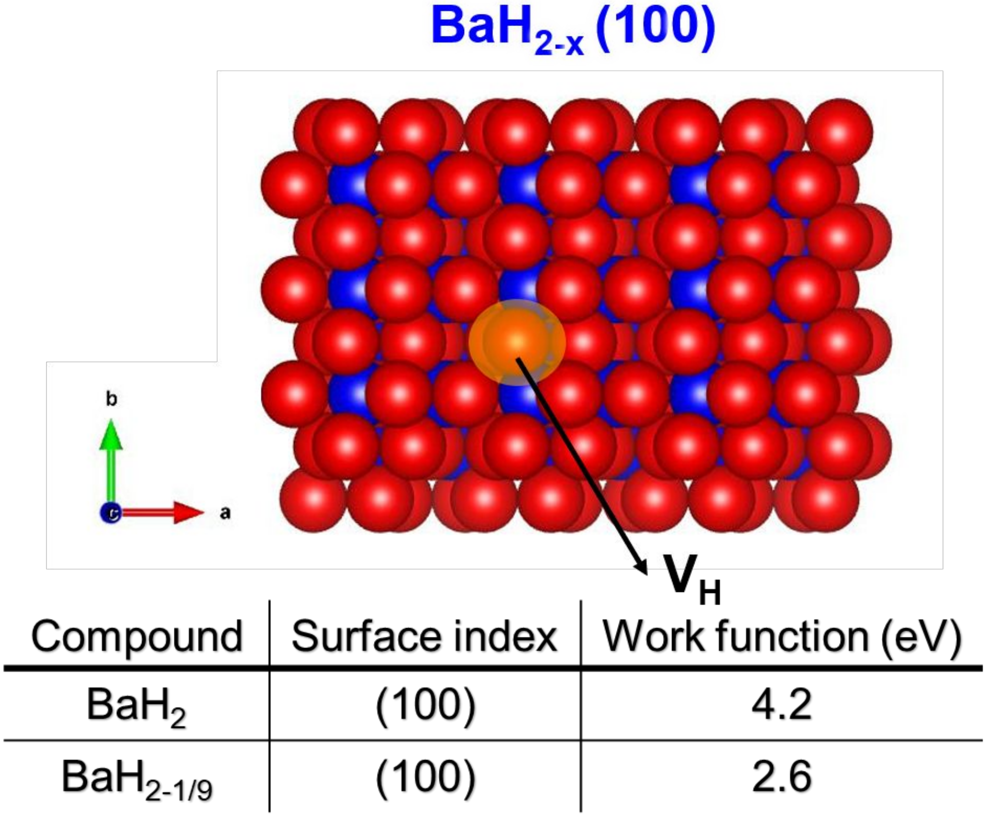

Compound	Surface index	Work function (eV)
BaH_2	(100)	4.2
$BaH_{2\text{-}1/9}$	(100)	2.6

Figure S5. DFT results for BaH_2 with H^- defects. Total energies and structural relaxations of BaH_2 with/without surface H^- anion defects were estimated from density functional theory (DFT) computation based on VASP first-principles code. We adopted the Perdew–Burke–Ernzerhof (PBE) exchange-correlation functional in DFT. The convergence criteria of energy and force were, respectively, 0.5×10^{-4} eV and 1.0×10^{-1} eV nm^{-1} for all models. The core electrons were handled with the projector augmented wave (PAW) method. The k-point mesh was created to keep a single k-point per 1/4 (nm^{-1}) in the reciprocal space. In BaH_2, (0 0 1), (0 1 0), (1 0 0), (0 1 1), (1 0 1), (1 1 0), and (1 1 1) surface models were relaxed using DFT, and the (1 0 0) surface was the most stable surface for BaH_2 (0.37 J m^{-2}). A notable feature in the computation is that a vacuum region of 2 nm is maintained in the unit cell.DFT calculations revealed that the (100) surface is the most stable in BaH_2. The work function of BaH_2 with H^- defects ($Ba^{2+}H^-_{(2-1/9)}e^-_{1/9}$) that trap electrons was estimated to be 2.6 eV.

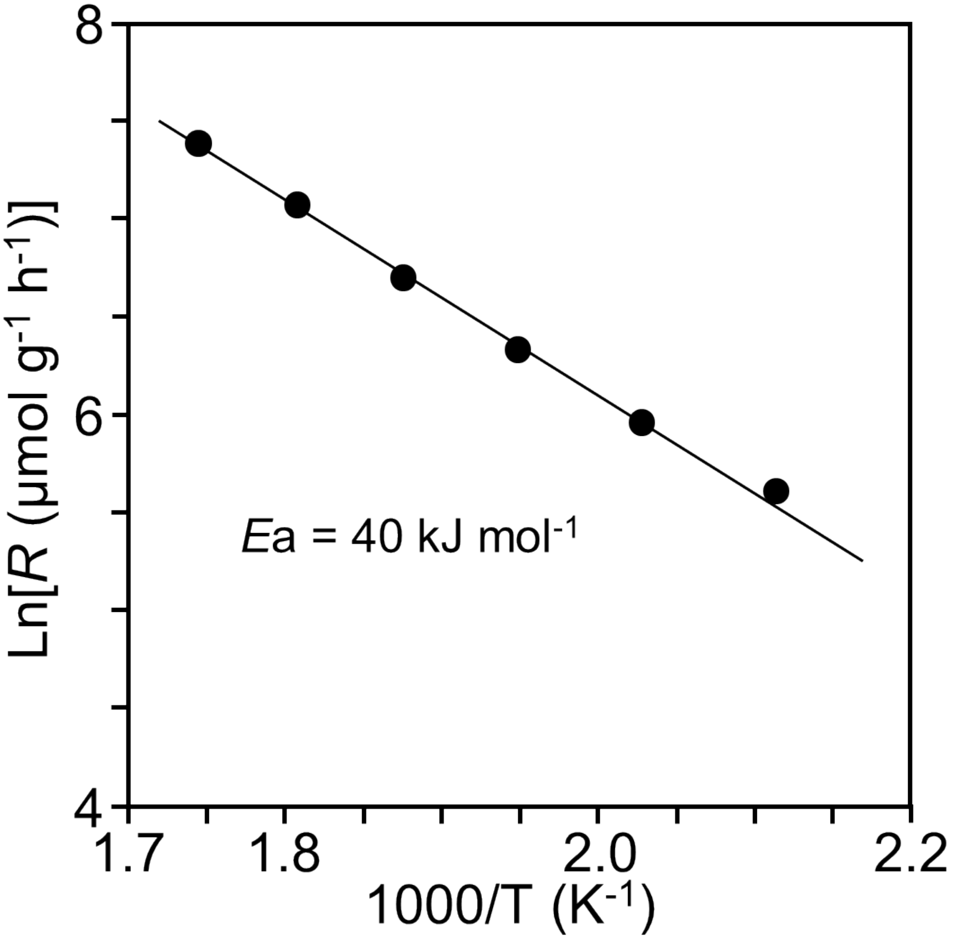

Figure S6. Arrhenius plots for ammonia synthesis activity of BaH_2-BaO/Fe/CaH_2 at 0.9 MPa. The apparent activation energy of BaH_2-BaO/Fe/CaH_2 was estimated to be 40±5 kJ mol^{-1}.

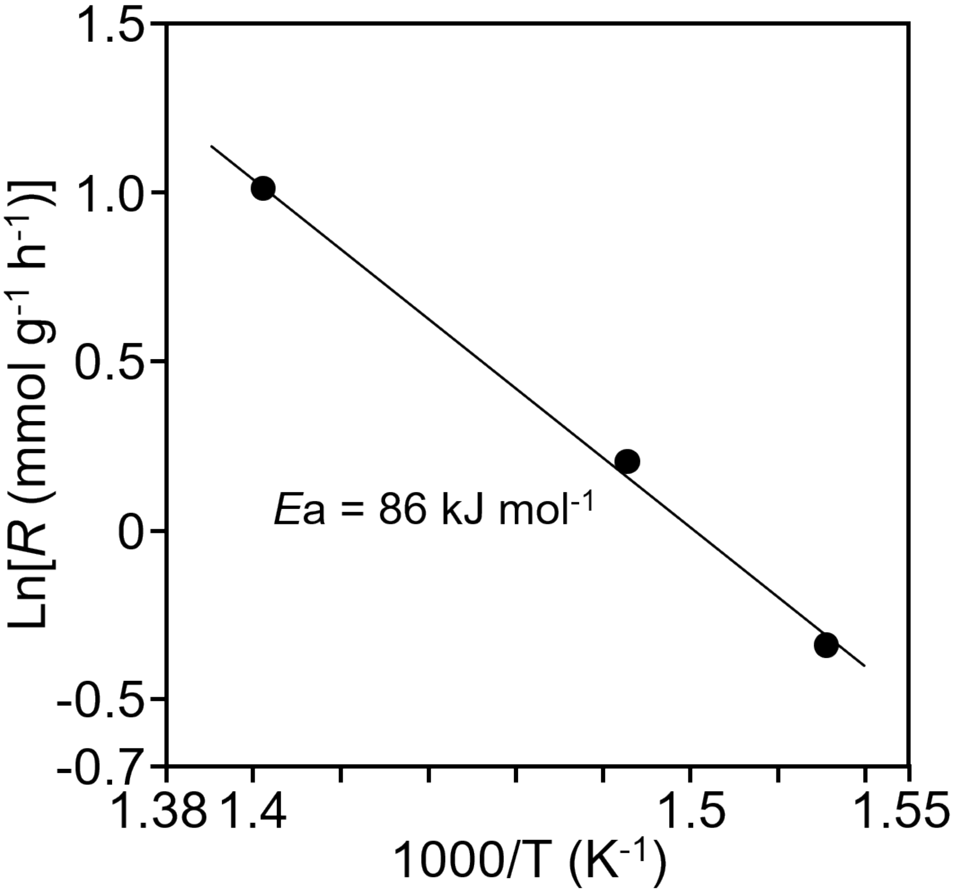

Figure S7. Arrhenius plots for $^{14}N_2$-$^{15}N_2$ isotropic exchange reaction over BaH_2-$BaO/Fe/CaH_2$. The apparent activation energy was about 86±5 kJ mol^{-1}.

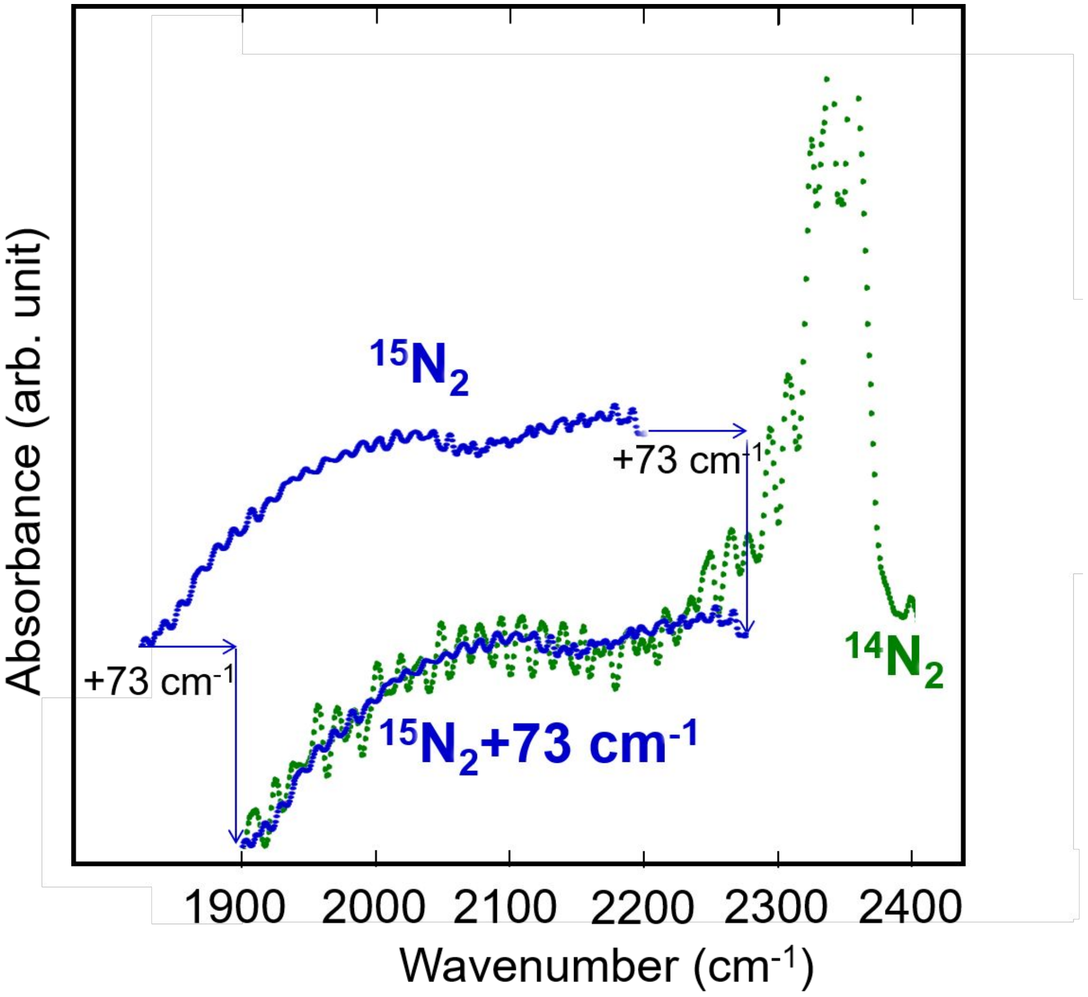

Figure S8. FT-IR spectra for $^{14}N_2$ and $^{15}N_2$ adsorbed BaH_2-BaO/Fe/CaH_2 (10 kPa of $^{14}N_2$ and $^{15}N_2$ at 25 °C). Spectrum "$^{15}N_2$+73 cm^{-1}" was obtained by blue-shifting the FT-IR spectrum for $^{15}N_2$ adsorbed BaH_2-BaO/Fe/CaH_2 (Spectrum "$^{15}N_2$") by 73 cm^{-1}. Spectrum $^{15}N_2$+73 cm^{-1} is consistent with the spectrum for $^{14}N_2$-adsorbed BaH_2-BaO/Fe/CaH_2 although the peaks observed in these spectra are weak and broad. Taking the isotope effect into account (ca. 2175 cm^{-1} × $(14/15)^{1/2}$), the broad peak below 2175 cm^{-1} can be attributed to the νN_2 band of N_2 adsorbed on BaH_2-BaO/Fe/CaH_2. One possible explanation for the broad band is that the electron-donating capability in the iron catalyst has a broad distribution. While the νN_2 band for BaH_2-BaO/Fe/CaH_2 is broad, the wavelength range is lower that of Ru/C12A7:e^-, which suggests that some of the electron-donating sites on BaH_2-BaO/Fe/CaH_2 have a stronger electron donating capability than those on Ru/C12A7:e^-.

Table S1. Catalytic activities of various transition metals for ammonia synthesis (300 °C).

Catalysts	S_{BET} (m^2 g^{-1})	Metal particle size (nm)	Pressure (MPa)	WHSV (mL g^{-1} h^{-1})	r_{NH_3} (mmol g^{-1} h^{-1})	Ref.
BaH_2-BaO/Fe/CaH_2	7	26.0	0.9	36000	5.5	This work
Promoted Fe	-	-	0.9	36000	5.4	5
			0.9	60000	9.0[a]	
			0.9	72000	10.8[a]	
Ru (10%)/BaH_2-BaO	20	4.0	0.9	36000	16.5	This work
Fe (10%)/BaH_2-BaO	15	3.5	0.9	36000	□	This work
Ru (10%)/Ba-$Ca(NH_2)_2$	101	2.7	0.9	36000	23.3	5
Cs-Ru (10%)/MgO	12	5.2	0.9	36000	0.6	5
Ru (2%)/C12A7:e^-	1	28.7	0.9	36000	0.8	5
Fe-LiH	16	29.6	1.0	60000	4.4	6
Co-LiH	43	27.6	1.0	60000	4.7	6
BaH_2-Co/CHTs	53	42.0	1.0	60000	4.8	7
Ru/$La_{0.5}Ce_{0.5}O_{1.75}$	42	1.7	1.0	72000	10.7	8
Ru/$La_{0.5}Pr_{0.5}O_{1.75}$	30	2.9	1.0	72000	8.9	9

[a]Ammonia formation rate estimated based on r_{NH_3} at the WHSV at 36000 mL g^{-1} h^{-1}.

Table S2. TOF for ammonia synthesis on various transition metal catalysts.

Catalysts	Temperature (°C)	Pressure (MPa)	TOF (s^{-1})	Ea (kJ mol^{-1})	Ref.
BaH_2-BaO/Fe/CaH_2[a]	300	0.9	12.2	40±5	This work
	200		1.7		This work
	100		0.2		This work
Ru (10%)/BaH_2-BaO[a]	300	0.9	$3.5x10^{-3}$	79±5	This work
	200		$3.2x10^{-4}$		This work
	100		-		This work
Ru (10%)/Ba-$Ca(NH_2)_2$[b]	300	0.9	$1.3x10^{-2}$	59	5
Cs-Ru (10%)/MgO[a]	300	0.9	$8.0x10^{-4}$	124	5
Ru/C12A7:e^-[a]	300	0.9	$5.6x10^{-2}$	49	5
Co/C12A7:e^-[b]	400	0.9	$9.3x10^{-3}$	50	10
Ni/LaN bulk[a]	400	0.9	0.17	60	11

[a]TOF calculated from the rate of ammonia synthesis divided by the number of surface N_s.
[b]TOF calculated from the rate of ammonia synthesis divided by the number of surface metal sites which was estimated based on the morphological mean particle sizes of transition metal particles (TEM image).

3. Refernces

1. Franzen, H. F.; Merrick, J.; Umana, M.; Khan, A. S.; Peterson, D. T. XPS spectra and crystalline potentials in alkaline-earth chalcogenides and hydrides. *J. Electron Spectrosc. Relat. Phenom.* **1977**, *11*, 439–443.
2. Koenig, M. F.; Grant, J. T. XPS studies of the chemical state of Ba on the surface of impregnated tungsten dispenser cathodes. *Appl. Surface Sci.* **1985**, *20*, 481–496.
3. Hattori, M.; Mori, T.; Arai, T.; Inoue, Y.; Sasase, M.; Tada, T.; Kitano, M.; Yokoyama, T.; Hara, M.; Hosono, H. Enhanced catalytic ammonia synthesis with transformed BaO. *ACS Catal.* **2018**, *8*, 10977–10984.
4. Mcintyre, N. S.; Zetaruk, D. G. X-ray photoelectron spectroscopic studies of iron oxides. *Anal. Chem.* **1977**, *49*, 1521–1529.
5. Kitano, M.; Inoue, Y.; Sasase, M.; Kishida, K.; Kobayashi, Y.; Nishiyama, K.; Tada, T.; Kawamura, S.; Yokoyama, T.; Hara, M.; Hosono, H. Self-organized ruthenium-barium core-shell nanoparticles on a mesoporous calcium amide matrix for efficient low-temperature ammonia synthesis. *Angew. Chem.* **2018**, *130*, 2678–2682.
6. Wang, P. K.; Chang, F.; Gao, W. B.; Guo, J. P.; Wu, G. T.; He, T.; Chen, P. Breaking scaling relations to achieve low-temperature ammonia synthesis through LiH-mediated nitrogen transfer and hydrogenation. *Nat. Chem.* **2017**, *9*, 64–70.
7. Gao, W. B.; Wang, P. K.; Guo, J. P.; Chang, F.; He, T.; Wang. Q.; Wu, G. T.; Chen, P. Barium hydride-mediated nitrogen transfer and hydrogenation for ammonia synthesis: a case study of cobalt. *ACS Catal.* **2017**, *7*, 3654–3661.
8. Ogura, Y.; Sato, K.; Miyahara, S.; Kawano, Y.; Toriyama, T.; Yamamoto, T.; Matsumura, S.; Hosokawa, S.; Nagaoka, K. Efficient ammonia synthesis over a $Ru/La_{0.5}Ce_{0.5}O_{1.75}$ catalyst pre-reduced at high temperature. *Chem. Sci.* **2018**, *9*, 2230–2237.
9. Ogura, Y.; Tsujimura, K.; Sato, K.; Miyahara, S.; Toriyama, T.; Yamamoto, T.; Matsumura, S.; Nagaoka, K. $Ru/La_{0.5}Pr_{0.5}O_{1.75}$ catalyst for low-temperature ammonia synthesis. *ACS Sustainable Chem. Eng.* **2018**, *6*, 17258–17266.
10. Inoue, Y.; Kitano, M.; Tokunari, M.; Taniguchi, T.; Ooya, K.; Abe, H.; Niwa, Y.; Sasase, M.; Hara, M.; Hosono, H. Direct activation of cobalt catalyst by $12CaO \cdot 7Al_2O_3$ electride for ammonia synthesis. *ACS Catal.* **2019**, *9*, 1670–1679.
11. Ye, T.-N.; Park, S.-W.; Lu, Y.; Li, J.; Sasase, M.; Kitano, M.; Tada, T.; Hosono, H. Vacancy-enabled N_2 actinvation for ammonia synthesis on an Ni-loaded catalyst. *Nature* **2020**, *583*, 391–407.
